# Neutral lipids as early biomarkers of cellular fate: the case of α-synuclein overexpression

**DOI:** 10.1038/s41419-020-03254-7

**Published:** 2021-01-07

**Authors:** Natalia P. Alza, Melisa A. Conde, Paola G. Scodelaro-Bilbao, Gabriela A. Salvador

**Affiliations:** 1 Instituto de Investigaciones Bioquímicas de Bahía Blanca (INIBIBB), Bahía Blanca, Argentina; 2 Departamento de Química–Universidad Nacional del Sur (UNS), Bahía Blanca, Argentina; 3 Consejo Nacional de Investigaciones Científicas y Técnicas (CONICET), Bahía Blanca, Argentina; 4 Departamento de Biología, Bioquímica y Farmacia-Universidad Nacional del Sur (UNS), Bahía Blanca, Argentina

**Keywords:** Biochemistry, Cell biology

## Abstract

α-synuclein (α-syn) accumulation and aggregation is a common pathological factor found in synucleinopathies, a group of neurodegenerative disorders that includes Parkinson´s disease (PD). It has been proposed that lipid dyshomeostasis is responsible for the occurrence of PD-related processes, however, the precise role of lipids in the onset and progression of neurodegenerative disorders remains unclear. Our aim was to investigate the effect of α-syn overexpression on neutral lipid metabolism and how this impacts on neuronal fate. We found lipid droplet (LD) accumulation in cells overexpressing α-syn to be associated with a rise in triacylglycerol (TAG) and cholesteryl ester (CE) levels. α-syn overexpression promoted diacylglycerol acyltransferase 2 upregulation and acyl-CoA synthetase activation, triggering TAG buildup, that was accompanied by an increase in diacylglycerol acylation. Moreover, the CE increment was associated with higher activity of acyl-CoA:cholesterol acyltransferase. Interestingly, α-syn overexpression increased cholesterol lysosomal accumulation. We observed that sterol regulatory element-binding protein (SREBP)-1 and SREBP-2 were differentially regulated by α-syn overexpression. The latter gave rise to a reduction in SREBP-1 nuclear translocation and consequently in fatty acid synthase expression, whereas it produced an increase in SREBP-2 nuclear localization. Surprisingly, and despite increased cholesterol levels, SREBP-2 downstream genes related to cholesterolgenesis were not upregulated as expected. Notably, phospholipid (PL) levels were diminished in cells overexpressing α-syn. This decrease was related to the activation of phospholipase A2 (PLA2) with a concomitant imbalance of the PL deacylation-acylation cycle. Fatty acids released from PLs by iPLA2 and cPLA2 action were esterified into TAGs, thus promoting a biological response to α-syn overexpression with uncompromised cell viability. When the described steady-state was disturbed under conditions favoring higher levels of α-syn, the response was an enhanced LD accumulation, this imbalance ultimately leading to neuronal death.

## Introduction

The biology of α-synuclein (α-syn) has attracted a great deal of interest since the association of this protein with a group of devastating neurodegenerative disorders named synucleinopathies, the most prevalent of which is Parkinson’s disease (PD). Genome-wide association studies disclose several genetic variants for the α-syn (SNCA) locus related both to idiopathic and inherited forms of PD, and other synucleinopathies^1,2^. A pathognomonic sign of these neurological disorders is the presence of intracellular inclusions called Lewy bodies whose main component is the α-syn protein. The formation process of Lewy bodies is still unclear but there is general consensus that overexpression and pathological aggregation of α-syn is one of the triggering factors^3,4^.

A large number of reports are available in the literature describing the complex biology of α-syn both under physiological and pathological conditions^5–7^. However, the role of α-syn in lipid homeostasis and the biological implications of this metabolic crosstalk for cellular fate is not well understood. Several structural biology studies demonstrate that α-syn has affinity for different kinds of lipids such as triacylglycerol (TAG) and cholesterol (chol). The protein also shows binding affinity for fatty acids (FAs) and phospholipids (PLs), these interactions being considered relevant to the induction of protein conformational changes, oligomerization, and fibrillation^8–14^.

Genetic network analysis has linked lipid dyshomeostasis to different processes involved in PD etiology^15^. The most striking evidence of α-syn involvement in the regulation of lipid metabolism came from in vivo studies using knock-out mice^16–20^. SNCA knock-out mice displayed altered polyunsaturated FA metabolism, with lower uptake of arachidonic acid observed in neurons and astrocytes and increased acylation of docosahexaenoic acid in PLs^21^. These findings shed light on adaptive responses triggered by α-syn silencing and highlight the role of the protein in FA turnover in the brain. Overexpression of α-syn triggers different cellular responses related to lipid metabolism and signaling. In our lab, we have previously demonstrated that α-syn overexpression induced the downregulation of phospholipase D1 and ERK1/2 signaling, thus triggering the loss of neuronal phenotype through the inhibition of neurofilament light chain expression^22^. In silico studies from the Yeger-Lotem lab, corroborated in yeast models, show the correlation between α-syn overexpression and several lipid metabolism-related genes^23^. One of the most interesting findings regarding neutral lipids is their accumulation of lipid droplets (LDs) and α-syn-LD binding properties^24,25^. LDs have been recently recognized as functional organelles residing in the cytosol and functioning as fat storage bodies with a very dynamic metabolism that senses cellular energy requirements^26,27^. LDs are composed of a neutral lipid core that mainly contains TAGs and cholesteryl esters (CEs) surrounded by a PL monolayer. This simple monolayer contains numerous proteins that act as sensors of metabolic fluxes and cell signaling, thus controlling lipid uptake, lysis and biosynthesis in LD^28,29^. In terms of α-syn biology and LDs, we have previously demonstrated that the overexpression of its A53T mutant form in dopaminergic neurons triggers increased acyl-CoA synthetase (ACS) activity and the accumulation of LDs, a response that is enhanced when cells are exposed to oxidative stress^30^. More recent lipidomic studies have shown that α-syn overexpression promotes the increase of stearoyl –CoA desaturase activity resulting in increased oleic acid (OA) cellular content and consequently LD accumulation in α-syn expressing cells^31^. Blockage of TAG formation by inhibiting de novo synthesis or stearoyl-CoA desaturase activity rendered neurons more vulnerable to α-syn overexpression and other injurious stimuli^30,31^. These findings argue in favor of a positive correlation between lipid dyshomeostasis and LD biogenesis as part of an adaptive response to α-syn overexpression and accumulation. It has been well demonstrated that in most cells exposed to nutrient withdrawal, oxidative stress and inflammatory stimuli, LD biogenesis and turnover are enhanced to provide protection against injury conditions^27,29,32,33^. However, precisely which metabolic changes are involved in LD formation in response to α-syn accumulation remains an open question. In this paper, we use human neuroblastoma cells to study the mechanisms involved in LD biogenesis and thus establish a link between α-syn overexpression and cellular fate. Here, we provide evidence that LDs coexist with α-syn in a steady-state without affecting cell viability, and that when this adaptive response is disrupted by conditions that increase α-syn expression, cell death is promoted.

## Material and methods

### Materials

Mouse monoclonal anti-DGAT2 (sc32399), rabbit monoclonal anti-α-synuclein (LB 509; sc-584809), rabbit monoclonal anti-SREBP-1 (sc-365513), mouse monoclonal anti-FAS (sc48357), mouse monoclonal anti-HMGCR (sc-271595), mouse monoclonal anti-β-actin (sc-47778), polyclonal horseradish peroxidase (HRP)-conjugated mouse anti-rabbit IgG (sc-2357), HRP-conjugated m-IgGκ (sc-516102), and normal anti-mouse IgG2A (sc-3878) were purchased from Santa Cruz Biotechnology, Inc. (Santa Cruz, CA, USA). Rabbit polyclonal anti-α-synuclein (cs-2642S) and rabbit polyclonal anti-LC3B (cs-2775) were purchased from Cell Signaling Technology (Beverly, MA, USA). Mouse monoclonal anti-α-tubulin (CP06) was purchased from EMD/Biosciences-Calbiochem (San Diego, CA, USA). Rabbit monoclonal anti-α-synuclein [MJFR1] (ab138501) and rabbit polyclonal anti-SREBP-2 (ab30682) were obtained from Abcam (Tecnolab, Bs. As., Argentina). Anti-LAMP-1 antibody [Alexa Fluor 647] (NB100-77683AF647) was obtained from Novus Biologicals (St. Louis, MO, USA). Alexa Fluor 546 goat anti-rabbit (A-11035) was purchased from Thermo Fisher (Invitrogen, CABA, Bs. As., Argentina). Cy-2-conjugated goat anti-rabbit (111-225-144) was obtained from Jackson Immunoresearch (Sero-immuno Diagnostics, GA, USA).

LysoTracker Red DND-99 (L7528), DAPI (D1306), SYTOX Green dye (S7020) and Nile Red (N1142) were from Molecular Probes (Eugene, OR, USA). TO-PRO-3 iodide (642/661) (T-3605), Hoechst (C-10339), Lipofectamine 2000 (11668019) and TRIZOL reagent (15596-026) were purchased from Thermo Fisher (Invitrogen, CABA, Bs. As, Argentina). Polyvinylidenedifluoride (PVDF) membranes were purchased from EMD Millipore (Millipore, Bedford, MA, USA). Dulbecco’s Modified Eagle Medium (DMEM) (52100047), trypsin (15090046) and antibiotic-antimycotic (15240062) were obtained from Gibco (USA). Geneticin (G418) (sc-29065), PLA2 inhibitors [arachidonoyl trifluoromethyl ketone (ATK, sc-201412B), bromoenol lactone (BEL, sc-201418), YM 26734 (sc-204410) and palmityl trifluoromethyl ketone (PTK, sc-201414)], and Oil Red O (sc-203749) were purchased from Santa Cruz Biotechnology, Inc. (Santa Cruz, CA, USA). Fetal bovine serum (FBS) was obtained from Natocor (Córdoba, Argentina). 3-(4,5-dimethylthiazol-2-yl)-2,5 diphenyltetrazolium bromide (MTT) (M2003), OA sodium salt (O7501), filipin complex from *Streptomyces filipinensis* (F9765), chloroquine (C6628), mevastatin (M2537) and DL-propranolol hydrochloride (P0884) were purchased from Sigma-Aldrich (St. Louis, MO, USA). Bortezomib (S1013) was obtained from Selleck Chemicals (Nuclilab.nl, Rotterdam, NL). Radiolabeled oleic acid [9,10-^3^H(N)] ([^3^H]-OA) (15–60 Ci/mmol) was purchased from New England Nuclear-Dupont (Boston, MA, USA.). TG color GPO/PAP AA (1780107) and enzymatic Colestat (1009802) were obtained from Wiener lab Group. Luna Universal Master Mix was from New England BioLabs. Manganese (II) chloride tetrahydrate (M5005-100G) was purchased from Sigma-Aldrich. All other chemicals used in the present study were of the highest purity available.

### Cell culture and transfection

The IMR-32 human neuroblastoma cell line was obtained from ATCC. Cells were transfected with the plasmid containing human wild type (WT) α-syn or the empty vector pcDNA3 using Lipofectamine 2000, following the manufacturer’s protocol. The selection was performed for 72 h post-transfection by the treatment with 400 µg/ml of G418. Stable transfected cells were cultured in a humidified atmosphere of 5% CO_2_ at 37 °C and grown in DMEM high-glucose medium supplemented with 200 µg/ml G418 and 10% (v/v) FBS. α-syn expression levels were checked by Western blot and immunocytochemistry. Cells were negative for mycoplasma.

### Experimental treatments

Cells (80–90% confluence) were treated with different drugs after replacement of DMEM with FBS-free medium. Inhibitors were further incubated for 24 h with the specified final concentration: 50 µM mevastatin, 100 µM DL-propranolol, 10 μM BEL, 50 μM ATK, 10 μM YM 26734, 45 µM PTK, 50 nM bortezomib, 50 µM chloroquine and 300 µM Mn. In OA exposure experiments, the medium was replaced by 0.5% bovine serum albumin (BSA) FA-free DMEM and cells were treated with 300–600 µM OA in the same medium overnight. Controls were treated with vehicle alone.

### Western blot

For the preparation of total cell extracts, IMR-32 cells stably expressing WT α-syn or the empty vector (pcDNA3) (1 × 10^7^ cells) were rinsed with PBS, scraped and centrifuged. The pellet was rinsed with PBS and resuspended in 80 μl of a lysis buffer [50 mM Tris pH 7.5, 150 mM NaCl, 0.1% Triton X-100, 1% NP-40, 2 mM EDTA, 2 mM EGTA, 50 mM NaF, 2 mM β-glycerophosphate, 1 mM Na_3_VO_4_, 10 μg/ml leupeptin, 5 μg/ml aprotinin, 1 μg/ml pepstatin, 0.5 mM PMSF, and 0.5 mM DTT]. Samples were incubated at 4 °C for 60 min, sonicated twice for 15 s, and centrifuged at 10,000 x *g* for 20 min. After the supernatant was decanted, protein concentration was determined using the Bradford method^34^. Cell lysates were mixed with Laemmli buffer 4x and samples with 25–50 μg of protein were separated by reducing 10% polyacrylamide gel electrophoresis and transferred into PVDF membranes^22^. Molecular weight standards (Spectra™ Multicolor Broad Range Protein Ladder, Thermo Scientific) were separated simultaneously. Membranes were blocked using 5% nonfat milk in TBS-T buffer for 1 h at room temperature. Then, membranes were exposed to primary antibodies (anti-α-syn, anti-HMGCR, anti-FAS, and anti-DGAT2) at 4 °C overnight, washed three times with TBS-T and subsequently incubated with the adequate HRP-conjugated secondary antibody for 1 h at room temperature. After membranes were washed three times with TBS-T, immunoreactive signals were detected through enhanced chemiluminescence using standard X-ray film and quantified by ImageJ2 (a freely available application in the public domain for image analysis and processing, developed and maintained by Wayne Rasband at the Research Services Branch, National Institute of Health, Bethesda, MD, USA)^35^.

### Dot blot

After treatments, cellular lysates were loaded onto nitrocellulose membranes and incubated with the A11 antibody (kindly provided by Dr. Charles Glabe). The A11 antibody recognizes conformational epitopes with specificity for prefibrillar oligomers^36^. Enhanced chemiluminescence with standard X-ray film was used for signal detection, which was quantified by ImageJ2.

### Immunofluorescence microscopy

Cells were grown onto glass coverslips and after treatments, fixed with 4% paraformaldehyde in PBS for 20 min. They were then permeabilized and blocked with 2% BSA in PBS and 0.1% Triton X-100 at room temperature for 45 min. For immunostaining, cells were incubated with the primary antibody (1:50 in PBS, 2% BSA, 0.1% Triton X-100) at room temperature for 2 h. Subsequently, cells were washed with PBS and exposed to the corresponding fluorescent secondary antibody (1:300 in PBS, 2% BSA) at room temperature for 1 h^22^. DAPI, Hoechst or TO-PRO-3 were used for nuclear staining. After washing with PBS and mounting, slides were observed with a Nikon Eclipse E-600 microscope or a confocal laser scanning microscope Leica DMIRE2. Image analysis was performed using the platform FIJI (at least 100 cells for each condition from two independent experiments).

### Oil Red O staining

After growth and fixation as described in the previous section, cells were washed with PBS three times and with water once. They were then exposed to a freshly prepared and filtered Oil Red O solution (570 μl of 0.5% Oil Red O in isopropanol and 380 μl of water) at room temperature for 1 h and rinsed thoroughly with water. For microscope analysis, nuclei were stained with DAPI and samples were mounted after washing^30^. Staining was assessed by bright-field and fluorescence microscopy and images were analyzed with FIJI platform. For spectrophotometric measurement, stained cells were incubated with isopropanol for 20 min to elute the dye and 2 aliquots of 200 μl were transferred to 96-well plated to measure the absorbance at 520 nm^37^. Results are expressed as AU per viable cell.

### Nile Red staining

Briefly, fixed cells were covered with a solution of 1.5 µg/ml of Nile Red in PBS and incubated at room temperature for 15 min. Nuclei were stained with Hoechst, then samples were washed with PBS and mounted^38^. Images were analyzed with FIJI platform.

### Filipin and lysotracker co-staining

Cells grown on coverslips were washed twice with PBS and incubated with 50 nM LysotrackerRed DND-99 in DMEM at 37 °C for 30 min^39^. Subsequently, cells were washed with PBS and fixed with 4% paraformaldehyde in PBS at room temperature for 20 min. After washing with PBS, cells were incubated with 100 µg/ml filipin at room temperature for 2 h^40^. Finally, samples were mounted and examined through fluorescence microscopy. Pearson’s correlation coefficients were calculated using Coloc2 software in ImageJ2 to determine the colocalization index.

### SYTOX Green staining

SYTOX Green is considered a marker of dead cells since it is impermeant to live cells but is able to permeate cells with loss of plasma membrane integrity. After cells were grown on coverslips and treated, they were incubated with SYTOX Green (2 nM) at 37 °C for 20 min. They were then washed three times with PBS and fixed with 4% paraformaldehyde^41^. Subsequently, nuclei were counterstained with DAPI. Coverslips were mounted and slides were observed using a fluorescence microscope.

### Measurement of TAG, chol, and CE

For intracellular total content of neutral lipids, cells were firstly subjected to lipid extraction following the method of Bligh and Dyer^42^. After lipid extracts were washed twice with Bligh and Dyer upper phase, dried under N_2_ and dissolved in chloroform/methanol (2:1, v/v), samples corresponding to 10 μg of lipid P were spotted on silica gel plates. Neutral lipids were separated by thin layer chromatography (TLC): silica gel G; hexane: diethyl ether (80:20, v/v). After separation, TAG, chol and CE spots were visualized using iodine vapor, scraped off the silica and eluted. The residue was resuspended in 100 μl of isopropanol, and concentrations of neutral lipids were determined using commercial kits (TG color GPO/PAP AA and enzymatic Colestat) following the manufacturer’s instructions.

### PL separation

To study PL composition, total lipids were extracted following the method of Bligh and Dyer^42^. After extraction, two-dimensional TLC was used for the separation of individual PLs^43^ (silica gel H; chloroform: methanol: ammonia (65:25:5, v/v) for the first dimension; chloroform: acetone: methanol: acetic acid: water (30:40:10:10:4, v/v) for the second dimension). Spots were visualized by exposure of the plate to iodine vapors and scraped off for lipid phosphorus determination.

### Lipid phosphorus measurement

Lipid phosphorus was determined according to the method described by Rouser and collaborators^43^. The determination was used in total lipid extracts in order to normalize lipid content and also in each spot after TLC resolution of individual PLs.

### Cell viability

The MTT reduction assay was used to assess cell viability. Briefly, cells were seeded at the density of 1 × 104 cells/well into 96-well plates. After 24 h, they were treated as specified above and incubated with MTT at a final concentration of 0.5 mg/ml for 2 h at 37 °C in a 5% CO_2_ atmosphere. The MTT-containing medium was removed and the formazan crystals were dissolved with 200 μl of 20% SDS (pH 4.7). The MTT reduction was measured spectrophotometrically at 570 nm^22^. Results are expressed as a percentage of the control.

### Quantitative RT-PCR

Total RNA was extracted using TRIZOL Reagent following the manufacturer’s instructions. After resuspension of RNA with nuclease-free water, the concentration was determined using a PicoDrop Spectrophotometer from A260:A280 absorbance ratio. Aliquots of 2 μg RNA were used to convert into cDNA using a High-Capacity RNA-to-cDNA™ kit (Applied Biosystems). RT-qPCR was performed using Luna Universal Master Mix and 0.2 μM of each primer. Gene expression was measured in a Rotor-Gene 6000 (Corbett Research, Australia). Ct values of ATG5, FAS, DHCR24 and DGAT2 mRNA from 3 different experiments were normalized according to the 2^−ΔΔCt^ method, using GAPDH as reference gene. Gene-specific primer sequences designed for RT-qPCR were to ATG5, forward: AGATGTGCTTCGAGATGTGTG and reverse: TCACTTTGTCAGTTACCAACGTC; FAS, forward: CTTCCGAGATTCCATCCTACGC and reverse: TGGCAGTCAGGCTCACAAACG; DHCR24, forward: GCCGCTCTCGCTTATCTTCG and reverse: GTCTTGCTACCCTGCTCCTT; DGAT2, forward: TGGTGCCCTACTCCAAGC and reverse: TGGTGTGGTACAGGTCGATG; GAPDH, forward: CGAGATCCCTCCAAAATCAA and reverse: TTCACACCCATGACGAACAT^44^.

### ACS activity assay

ACS activity was determined in cellular homogenates^45^. In brief, 1 × 106 cells were washed with PBS, scraped and centrifuged. Cells were transferred to a homogenizer after the addition of 200 μl of a buffer containing 20 mM MgCl_2_, 10 mM ATP, 1 mM CoA, 1 mM 2-mercaptoethanol, 100 mM Tris-HCl (pH 8.0). An aliquot of 150 μl of the homogenate was incubated with [^3^H]-OA (0.2 μCi, 50 μM) at 37 °C for 15 min. The reaction was stopped by the addition of 2.25 ml isopropanol:heptane:1 M H_2_SO_4_ (40:10:1 v/v/v) and subsequently 1.5 ml heptane and 1 ml water were added. After washing the aqueous phase with 2 ml heptane containing 4 mg/ml OA, the radioactivity was measured by liquid scintillation counting. Results are expressed as dpm of [^3^H]-oleoyl-CoA per μg of protein.

### TAG lipase activity assay

For the determination of TAG lipase activity, cellular homogenates (1 × 106 cells) were incubated with [^3^H]-Glycerol-TAG (350,000 dpm/μmol) in 20 mM phosphate buffer (pH 7.0) at 37 °C for 30 min^46^. Then, 5 ml chloroform:methanol (2:1, v/v) was added to stop the reaction. After the addition of 1 ml 0.05% CaCl_2_ and centrifugation, the aqueous phase containing [^3^H]-glycerol was collected in vials for counting by liquid scintillation. For the separation of [^3^H]-glycerol monoacylglycerol (MAG), [^3^H]-glycerol diacylglycerol (DAG) and [^3^H]-glycerol-TAG, TLC of the organic phase was performed using the appropriate standards (silica gel G; hexane:diethyl ether:acetic acid 50:50:2.6, v/v). Spots were visualized with iodine vapors and scraped into scintillation vials for liquid scintillation counting. Results are expressed as dpm per μg of protein.

### Acyl-CoA:cholesterol acyltransferase (ACAT) activity assay

The enzyme activity was assessed by the measure of the synthesis of [^3^H]-cholesteryl oleate^47^. After 24 h of starvation, cells (1 × 106) were incubated with [^3^H]-OA (0.5 μCi/dish) in FBS-free medium containing OA (1.5 μM) and lipid-free BSA (4 mol oleic acid/mol BSA) for 4 h at 37 °C. After lipid extraction and TLC separation (silica gel G; hexane: diethyl ether, 80:20, v/v), the formation of [^3^H]-cholesteryl oleate was measured by liquid scintillation counting.

### [^3^H]-OA incorporation into lipids

After treatments, cells (1 × 106) were incubated for 24 h at 37 °C with FBS-free DMEM containing: [^3^H]-OA (0.5 μCi/dish), OA (1.5 μM) and lipid-free BSA (4 mol oleic acid/mol BSA) in order to incorporate [^3^H]-OA^48^. Then, cells were washed three times with PBS, scraped off and the lipids extracted. Lipid extracts were subjected to TLC for the separation of free FA (FFA), MAGs, DAGs, and TAGs (silica gel G, hexane:diethyl ether:acetic acid 70:30:2.6, v/v) and PLs (above-described chromatographic conditions). Lipids were visualized by exposure to iodine vapors, and then scraped off the plate into vials. Radioactivity was determined in a liquid scintillation spectrometer. The incorporation of [^3^H]-OA into MAGs, DAGs, TAGs and PLs is expressed as dpm per μM individual PL or percentage of the control.

### Statistical analysis

The data were summarized as means ± SD based on three independent experiments and analyzed using GraphPad Prism 5.00 software (GraphPad Software, Inc., La Jolla, CA, USA). The variance similarity was determined by the *F*-test and variances were not significantly different between groups. Statistical significance was determined by a two-tailed unpaired Student’s *t*-test for two-group samples or one-way ANOVA followed by a Tukey’s test for multi-group samples. *p*-values lower than 0.05 were considered statistically significant; *, **, or *** represent *p* < 0.05, *p* < 0.01, and *p* < 0.001 respectively.

## Results

### WT α-syn overexpression alters cellular lipid homeostasis

**Fig. 1:**
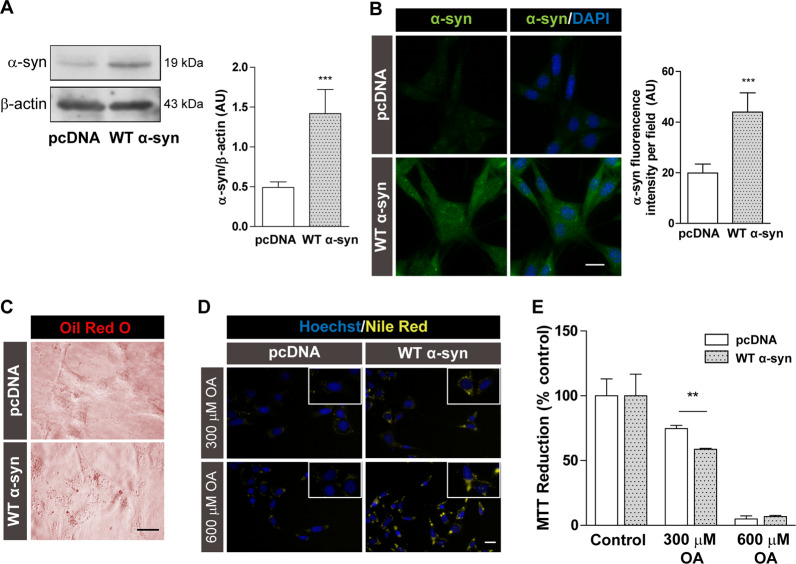
α-syn overexpression promotes LD accumulation. **A**, **B** α-syn expression levels in IMR-32 neuroblastoma cells stably transfected with the empty vector (pcDNA) or the α-syn plasmid expressing human wild type α-syn (WT α-syn) using Western blot and immunocytochemistry studies. DAPI was used as nuclear marker. **C** Microscopic visualization of LDs through Oil Red O staining in the neuronal model of α-syn overexpression. **D** Nile Red staining showing an increase in the amount and size of LDs in cells overexpressing α-syn treated with 300–600 µM OA, compared to control cells. Hoechst was used as nuclear marker. **E** Effect on cell viability after 300–600 µM OA treatment. **A**–**E** *Scale bars* 20 µm. All experiments were repeated three times. Bars represent means ± standard deviation (SD, *n* = 3). **p < 0.01, ***p < 0.001 with respect to the control conditions.

Our lab has previously established that PD-related insults, such as iron overload and overexpression of A53T α-syn mutant, shift neuronal lipid metabolism towards LD accumulation^30,49^. In the present work, our aim was to investigate whether α-syn alters lipid homeostasis in a neuronal model of WT α-syn overexpression. For this purpose, we used the human neuroblastoma cell line IMR-32 either stably transfected with pcDNA3 vector containing human WT α-syn (WT α-syn) or the empty vector (pcDNA) as transfection control. This cell line with stable transfection was able to express 200% of α-syn compared to empty vector controls (Fig. 1A, B). Working with the same experimental model, these levels of WT α-syn overexpression were able to induce the downregulation of phospholipase D1 and consequent cytoskeleton alterations. Thus, this biological model can be useful for the study of early stages of α-syn-associated neurodegeneration^22^.

The first sign of lipid homeostasis impairment mediated by α-syn overexpression was the accumulation of LDs. Other authors previously reported evidence that α-syn displayed the same effect in yeast, rat cortical neurons, and human iPSC-derived neurons^25,31^. Through Oil Red O staining, we observed an increase in LD formation in cells overexpressing WT α-syn (Fig. 1C). To reinforce this data, LD formation was induced by OA treatment and detected using Nile Red staining. While the treatment with 300 µM OA increased the number of LDs in WT α-syn cells, exposure to 600 µM OA enlarged the LD size (Fig. 1D). Interestingly, cells overexpressing WT α-syn were more sensitive to 300 µM OA than pcDNA cells (Fig. 1E), suggesting that an excess of exogenous FA disrupted the balance and enhanced α-syn toxicity. Viability assays determined that 600 µM OA exposure was very toxic for control and WT α-syn cells (Fig. 1E), indicating that the threshold of OA incorporation into TAG was overpassed in both cell types.

**Fig. 2:**
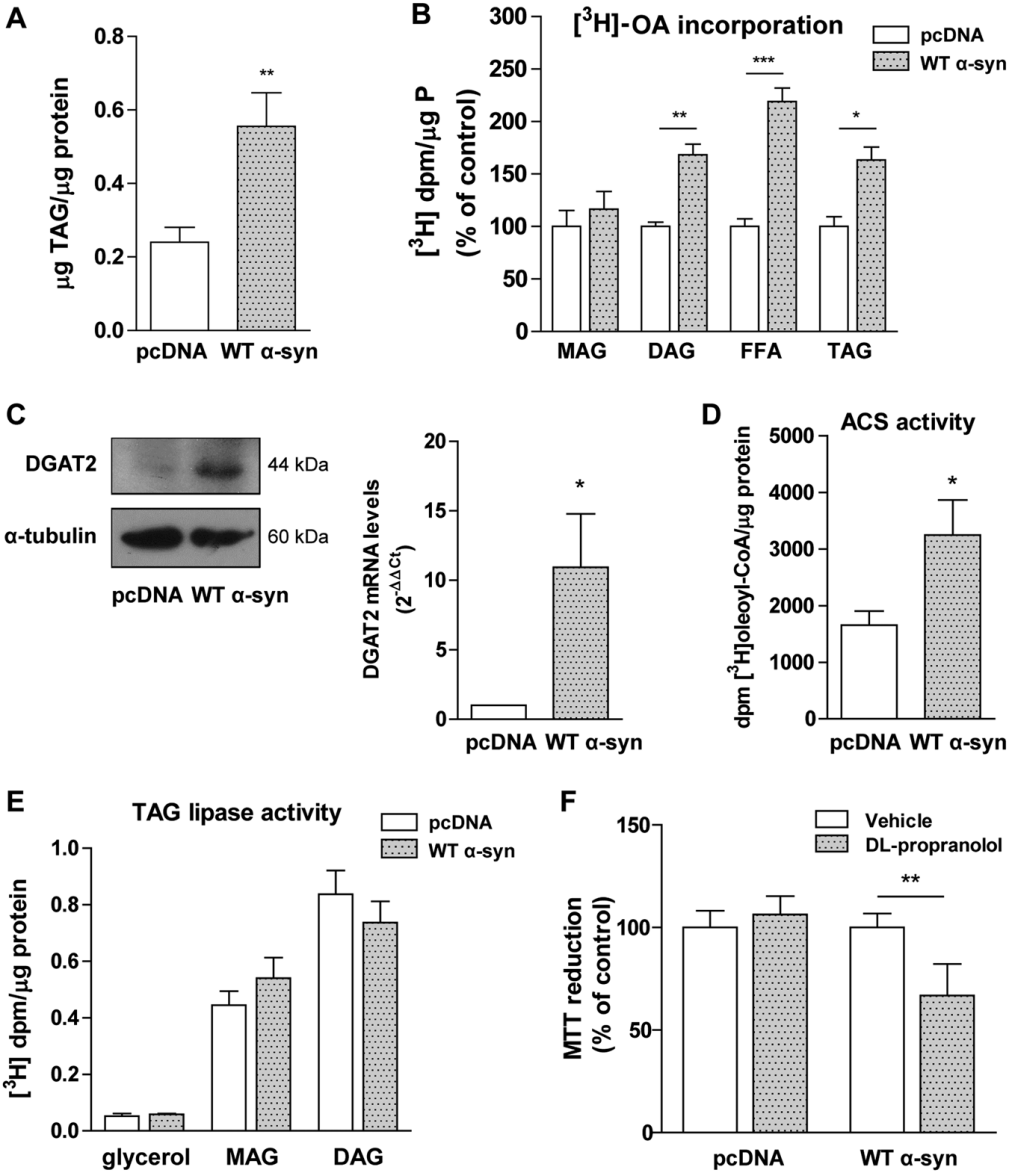
Impairment in TAG biosynthesis protects against α-syn overexpression. **A** Cellular TAG content was measured with a detection kit in pcDNA and WT α-syn cells. **B** [^3^H]-OA incorporation into MAG, DAG and TAG, and FFA release were measured in our model. **C** DGAT2 upregulation in cells overexpressing α-syn was determined by Western blot and RT-qPCR. **D** ACS activity was studied by the measurement of [^3^H] oleyl -CoA formation in pcDNA and WT α-syn neurons. **E** TAG lipase activity was determined through the detection of the products of TAG hydrolysis in a radioactivity assay. **F** The role of TAG biosynthesis during α-syn overexpression was determined in cells treated with the lipin-1 inhibitor DL-propranolol by the MTT reduction assay. **A**–**F** All experiments were repeated three times. Bars represent means ± standard deviation (SD, *n* = 3). *p < 0.05, **p < 0.01, ***p < 0.001 with respect to the control conditions.

The LD core is mainly composed of TAGs and CEs. Total cellular TAG content was augmented by α-syn overexpression (100% increase) as shown in Fig. 2A. In agreement, [^3^H]-OA pre-labeled cells overexpressing α-syn presented higher FA incorporation into DAGs and TAGs than control cells (Fig. 2B). Radiolabeling assays also showed a higher FFA release and no changes in MAG acylation in WT α-syn cells (Fig. 2B). To further evaluate TAG synthesis, we analyzed the expression of diacylglycerol acyltransferase 2 (DGAT2). Both mRNA and protein levels of DGAT2 were upregulated in WT α-syn cells (Fig. 2C). To confirm the increased FA acylation observed in cells overexpressing α-syn, the activity of ACS was measured. WT α-syn cells displayed higher ACS-specific activity, implying an increase in FA availability and priming for acylation (Fig. 2D). Given that TAG content is regulated by a balance between acylation and hydrolysis processes, we then measured TAG lipase activity using [^3^H]-glycerol-TAG as substrate. The formation of [^3^H]-glycerol, [^3^H]-glycerol MAG and [^3^H]-glycerol DAG was not altered by WT α-syn overexpression (Fig. 2E), allowing us to speculate that the increased TAG formation in WT α-syn cells was produced by an imbalance between acylation and deacylation processes. To evaluate the role of TAG accumulation during WT α-syn overexpression, cells were treated with DL-propranolol, a pharmacological inhibitor of lipin-1, which is the rate limiting enzyme in TAG de novo biosynthesis (Kennedy pathway). Cell viability was differentially affected by the presence of DL-propranolol, WT α-syn cells being more sensitive to the inhibition of lipin-1 than control cells (Fig. 2F).

**Fig. 3:**
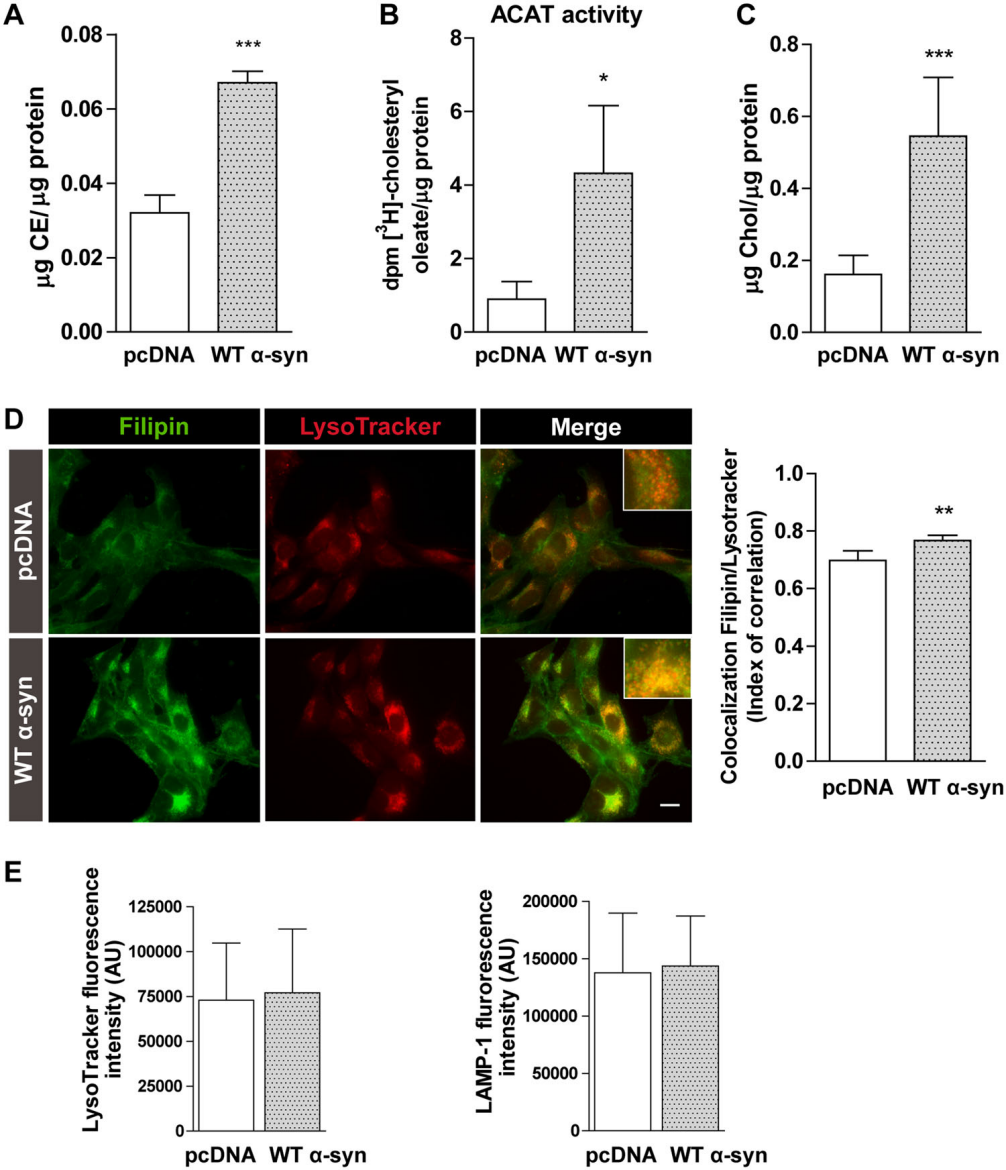
α-syn overexpression alters chol homeostasis. **A** CE content was determined by a detection kit in pcDNA and WT α-syn cells. **B** ACAT activity was measured in our model by the incorporation of [^3^H]-OA in chol. **C** Cellular chol was measured using a detection kit. **D** Chol stained with filipin co-localized with the lysosomal probe Lysotracker in cells overexpressing α-syn. **E** Quantification of fluorescence intensity of lysosomal markers, Lysotracker and LAMP-1. All experiments were repeated three times. **A**–**E** *Scale bars* 20 µm. All experiments were repeated three times. Bars represent means ± standard deviation (SD, *n* = 3). *p < 0.05, **p < 0.01, ***p < 0.001 with respect to the control conditions.

The other class of neutral lipids potentially stored in LDs is CEs. We found that total CE levels were also augmented in WT α-syn cells (100% increase) (Fig. 3A). The synthesis of CEs could be consistent with the activation of ACAT, the enzyme responsible for the conversion of free chol to CE. Upon labeling cells with [^3^H]-OA to monitor the esterification of chol, we found higher activation of ACAT in cells overexpressing WT α-syn (Fig. 3B). When we analyzed the free chol content in our model, we detected that WT α-syn cells contained more unesterified free chol (Fig. 3C). Moreover, WT α-syn cells displayed higher fluorescence intensity with the specific free chol probe filipin. Staining experiments also revealed that filipin co-localized with the lysosomal marker Lysotracker to a greater extent in WT α-syn cells (Fig. 3D). The described pattern points towards the sequestration of chol in lysosomes under our experimental conditions. LAMP-1 was used as an additional lysosomal marker showing that the organelle distribution (data not shown) and the fluorescence intensity did not change in WT α-syn cells, coincidently with the results obtained with Lysotracker (Fig. 3E).

### SREBPs are involved in lipid dyshomeostasis triggered by WT α-syn

**Fig. 4:**
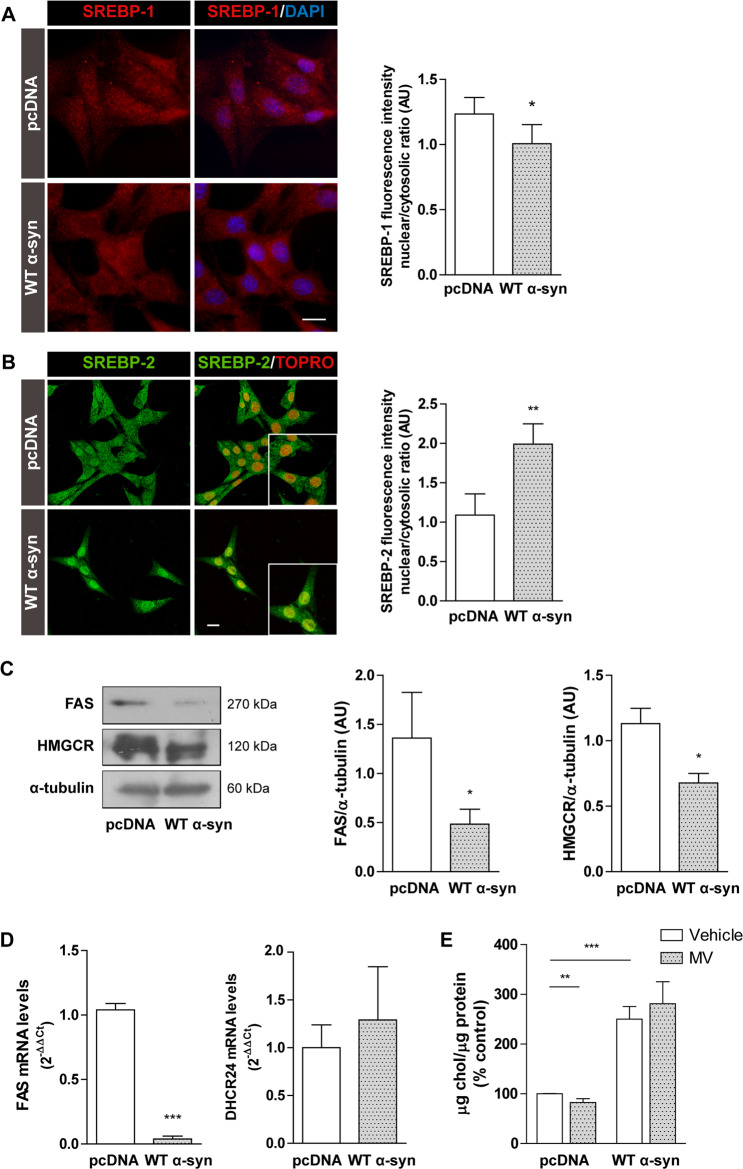
SREBP status and downstream gene targets change in cells overexpressing α-syn. **A**, **B** SREBP-1 and SREBP-2 nuclear translocation was determined by immunocytochemistry studies. DAPI or TO-PRO-3 were used as nuclear markers. **C**, **D** Expression levels of the downstream gene targets FAS, HMGCR and DHCR24 were measured by western blot and/or RT-qPCR. **E** Chol content upon MV treatment was determined using a commercial kit. **A**–**E** *Scale bars* 20 µm. All experiments were repeated three times. Bars represent means ± standard deviation (SD, *n* = 3). *p < 0.05, **p < 0.01, ***p < 0.001 vs control.

In order to further characterize the mechanisms involved in α-syn-induced LD accumulation, we studied the status of the master regulators of lipid homeostasis: sterol regulatory element-binding protein (SREBP)-1 and SREBP-2. SREBP-1 exists as two isoforms: SREBP-1a and SREBP- 1c, which are responsible for directly enhancing gene transcription related with FA, TAG and PL synthesis^50^. SREBP-2 mainly regulates chol metabolism^50^. Taking into account that the activation of SREBPs implies the nuclear translocation of the mature form after proteolytic cleavage, we checked the cellular localization of the transcription factors. Whereas SREBP-1 nuclear localization was slightly diminished in WT α-syn cells (20% reduction of nuclear/cytosolic ratio), SREBP-2 nuclear translocation was induced by WT α-syn overexpression (80% increase in the nuclear/cytosolic ratio) (Fig. 4A, B).

Next, we correlated these findings with the expression of SREBP downstream genes through Western blot and RT-qPCR. Both protein and mRNA levels of FA synthase (FAS), which depends on SREBP-1c activity, were diminished by WT α-syn overexpression (65% and 95%, respectively) (Fig. 4C, D), in agreement with the lower activation of SREBP-1.

With respect to SREBP-2 nuclear translocation, two enzymes that participate in chol synthesis were paradoxically not upregulated by α-syn overexpression. WT α-syn cells showed a 40% decrease in 3-hydroxy-3-methylglutaryl-CoA reductase (HMGCR) levels (Fig. 4C) while no changes were observed in 24-dehydrocholesterol reductase (DHCR24) mRNA levels (Fig. 4D). To ascertain if increased chol levels were due to HMGCR enzyme activation, we used the selective enzyme inhibitor mevastatin (MV). Whereas WT α-syn contained the same levels of chol upon treatment with MV, control cells were sensitive to the inhibitor, displaying lower lipid levels (20% decrease) (Fig. 4E).

Recent studies have proposed autophagy genes as SREBP-2 targets^51,52^, autophagy related 5 (ATG5) being one of these. We observed that ATG5 was upregulated by α-syn overexpression (Supplementary Fig. S1A). Moreover, the blockage of autophagy through chloroquine (CQ) treatment displayed higher expression of the autophagosome marker LC3B in WT α-syn cells (200% increase) (Supplementary Fig. S1B). Interestingly, when autophagy was blocked, neutral lipid accumulation was enhanced in WT α-syn cells (Supplementary Fig. S1C), inferring that lipophagy played a role in α-syn-induced LD accumulation. Indeed, autophagy blockage only reduced the viability of WT α-syn cells, without affecting pcDNA cells (Supplementary Fig. S1D).

### Membrane remodeling is responsible for neutral lipid accumulation during α-syn overexpression

**Fig. 5:**
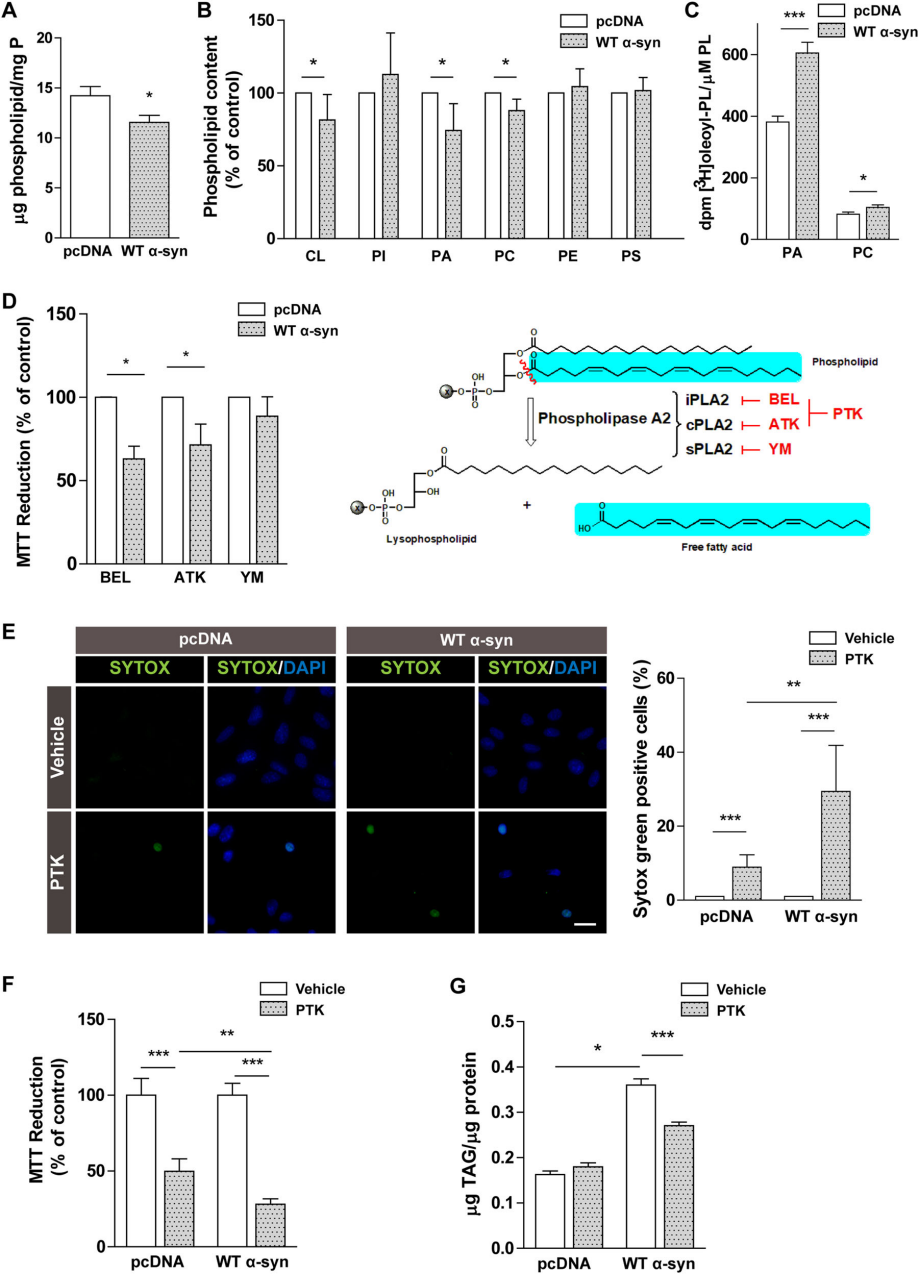
PL deacylation-acylation cycle imbalance causes TAG accumulation, protecting against α-syn overexpression. **A** Reduction of total lipid phosphorus in cells overexpressing α-syn was determined by the method of Rouser. **B** PL profiles (CL cardiolipin, PI phosphatidylinositol, PA phosphatidic acid, PC phosphatidylcholine, PE phosphatidylethanolamine, PS phosphatidylserine) of pcDNA and WT α-syn cells were analyzed by TLC separation followed by lipid phosphorus determination. **C** [^3^H]-OA incorporation into PA and PC was detected in our model. **D** The effect of the activity of different PLA2 isoforms on cell viability was determined by the treatment with specific inhibitors: BEL for iPLA2 inhibition, ATK for cPLA2 inhibition and YM for sPLA2 inhibition. **E**, **F** iPLA2 and cPLA2 activation role during α-syn overexpression was determined through SYTOX Green staining and the MTT reduction assay using the dual inhibitor PTK. DAPI was used as nuclear marker. **G** The involvement of α-syn-induced PLA2 activation in TAG accumulation was assessed by TAG measurement under iPLA2 and cPLA2 inhibition by PTK in pcDNA and WT α-syn neurons. The schematic view shows the action of PLA2 on PL hydrolysis at the sn-2 position and isoform-specific inhibitors BEL, ATK and YM, and PTK, a dual inhibitor of iPLA2 and cPLA2. **A**–**G** *Scale bars* 20 µm. All experiments were repeated three times. Bars represent means ± standard deviation (SD, *n* = 3). *p < 0.05, **p < 0.01, ***p < 0.001 vs. control.

Taking into account that FAS levels were downregulated, we sought to establish additional FA sources for the acylation of neutral lipids stored in LDs during α-syn overexpression. We then tested whether PL deacylation could be responsible for the higher FA bioavailability. We analyzed total PL content and under our experimental conditions found lower levels in cells overexpressing WT α-syn (Fig. 5A). An analysis of the distribution of PL classes showed that phosphatidylcholine (PC), cardiolipin (CL), and phosphatidic acid (PA) levels were diminished in WT α-syn cells (15%, 20%, and 25% decrease, respectively) (Fig. 5B). We also observed a rise in the incorporation of [^3^H]-OA into PA and PC in WT α-syn cells (59% and 28% increase, respectively), thus suggesting an active FA turnover in PL during α-syn overexpression (Fig. 5C).

Based on these results, we hypothesized that LD accumulation triggered by α-syn might be related to a higher activation of PL deacylation pathways. To test this hypothesis, we firstly evaluated the effect of inhibiting PLA2 activation on cell viability. There are several PLA2 isoforms that catalyze the hydrolysis of the FA at the sn-2 position of PLs to yield FFA and lysophospholipids (LPLs). Hence, pcDNA and WT α-syn cells were exposed to isoform-specific PLA2 inhibitors in order to assess the role of iPLA2, cPLA2 and sPLA2 using the MTT reduction assay. We determined that the inhibition of iPLA2 by BEL and cPLA2 by ATK, but not sPLA2 by YM, reduced the viability of cells overexpressing α-syn (Fig. 5D). In line with these findings, the blockage of both iPLA2 and cPLA2 by PTK caused a 200% rise in nuclei-positive cells in WT α-syn neurons in staining experiments using the cell death probe SYTOX Green (Fig. 5E). Furthermore, cells challenged with PTK showed diminished viability, this effect being more pronounced in WT α-syn cells (Fig. 5F). Lastly, to ascertain whether TAG accumulation is linked to iPLA2 and cPLA2 activation, lipid levels were evaluated in the presence of PTK (a dual inhibitor of both isoforms). Lipid analysis revealed reduced TAG content in WT α-syn cells treated with PTK (Fig. 5G). These data suggest that membrane remodeling catalyzed by iPLA2 and cPLA2 is a necessary event for LD formation during α-syn overexpression. Altogether, our results demonstrate that α-syn triggered an imbalance in the PL deacylation-acylation cycle that finally impacted TAG accumulation as a protective strategy.

### Enhancers of α-syn accumulation trigger LD formation and decrease cell viability

**Fig. 6:**
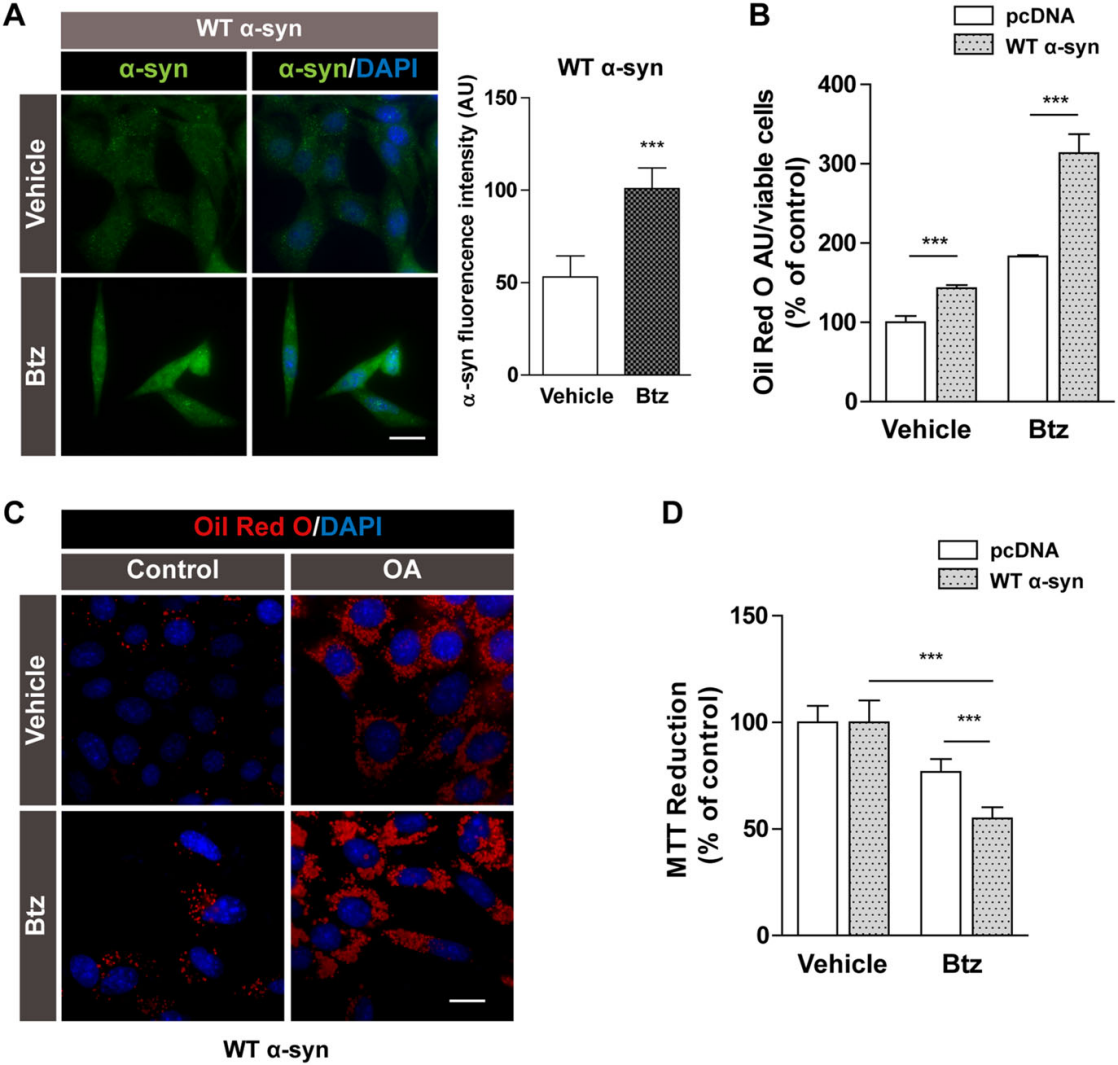
Btz treatment rendered neurons more vulnerable to α-syn expression. **A** Immunocytochemistry studies show the increased expression levels of α-syn under proteasome blockage by Btz in WT α-syn cells. DAPI was used as nuclear marker. **B**, **C** The effect of Btz treatment on neutral lipid accumulation was assessed by spectrophotometric measurement of Oil Red O in pcDNA and WT α-syn cells and microscopic visualization of Oil Red O staining under OA exposure of cells overexpressing α-syn. DAPI was used as nuclear marker. **D** Neuronal fate changes induced by Btz treatment were determined by the MTT reduction assay. **A**–**D** *Scale bars* 20 µm. All experiments were repeated three times. Bars represent means ± standard deviation (SD, *n* = 3). ***p < 0.001 vs. control.

To investigate the far-reaching effects of α-syn-induced impairment of lipid homeostasis on neuronal fate, we challenged WT α-syn cells with agents that increase protein levels. First, we exposed WT α-syn cells to bortezomib (Btz) in order to block proteosomal degradation. Cells treated with Btz expressed higher levels of α-syn (Fig. 6A). To determine whether the rise in α-syn induced by Btz influenced neutral lipid content, cells were stained with Oil Red O. Btz treatment caused a buildup of neutral lipids both in pcDNA and WT α-syn cells compared to controls treated with vehicle alone (80% and 150% increase, respectively) (Fig. 6B). In keeping with these findings, the potentiation of α-syn-induced LD accumulation exhibited by WT α-syn cells after proteasome inhibition was even more marked during OA exposure (Fig. 6C). Cells overexpressing α-syn were more sensitive to Btz than control cells owing to the more pronounced reduction in cell viability after treatments (Fig. 6D).

**Fig. 7:**
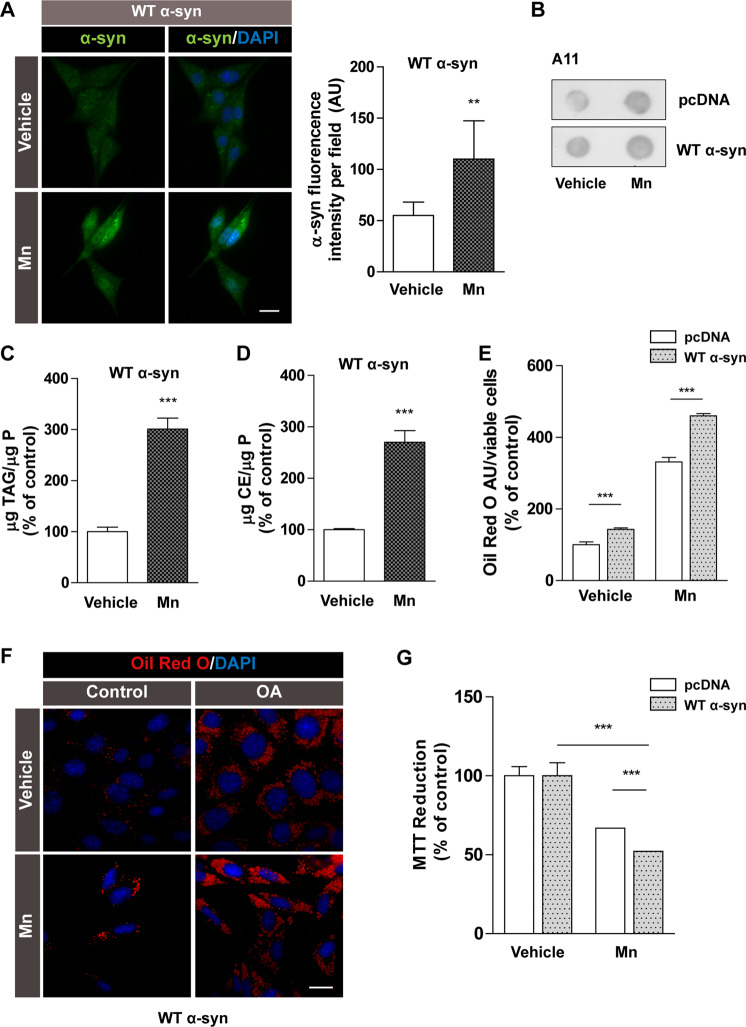
The presence of Mn rendered WT α-syn cells more sensitive, affecting cell viability. **A** Increased α-syn levels in WT α-syn exposed to Mn were detected by immunocytochemistry. DAPI was used as nuclear marker. **B** The enhancement of α-syn aggregation under Mn exposure was determined by Dot blot using A11 antibody. **C**, **D** TAG and CE content during Mn treatment was analyzed through detection kits in WT α-syn cells. **E**, **F** LD accumulation during Mn exposure was assessed trough Oil Red O staining. DAPI was used as nuclear marker. **G** The MTT reduction assay was used to evaluate the effect of Mn treatment on cell viability. **A**–**G** *Scale bars* 20 µm. All experiments were repeated three times. Bars represent means ± standard deviation (SD, *n* = 3). ***p < 0.001 vs control.

We then challenged cells with Mn as the metal is a well-described promoter of α-syn expression and aggregation^53^. We observed augmented protein levels in Mn-treated cells (Fig. 7A). This was accompanied by enhancement of α-syn aggregation detected through Dot blot using the conformational antibody A11 that recognizes oligomeric aggregates^36^ (Fig. 7B). WT α-syn cells exposed to Mn presented a buildup of TAG and CE (300% and 270%, respectively) (Fig. 7C, D). We also reinforced this data with Oil Red O staining experiments that showed an exacerbation of α-syn-induced LD accumulation after Mn treatment (Fig. 7E, F). In agreement with the results obtained with Btz-challenged cells, the overexpression of α-syn rendered cells more vulnerable to Mn exposure (Fig. 7G).

## Discussion

Lipid metabolism imbalance has been considered a common feature in neurodegenerative diseases such as PD^15,54^. In the present work we sought to shed light on undisclosed aspects of lipid metabolism in the presence of α-syn. Through a cellular model of α-syn overexpression, we establish that α-syn impairs lipid homeostasis and has a decisive impact on neuronal fate. We demonstrate that WT α-syn alters neutral lipid metabolism, culminating in LD accumulation. These organelles, specialized in neutral lipid storage, are considered to be managers of cellular stress response during infection, cancer, neurodegeneration and other disorders, by preventing FA-induced toxicity^29,33^. Several studies also found that α-syn expression promotes the formation of LD in yeast, rat cortical neurons, and human iPSC-derived neurons^25,31^. Furthermore, a previous work of our group showed that the overexpression of A53T α-syn mutant in dopaminergic neurons induces LD accumulation, which is potentiated under iron exposure^30^. Besides the above-mentioned induction of LD biogenesis, α-syn interacts with LD membrane^24,55^. Cumulative evidence supports the ability of α-syn to bind PLs and FAs. These interactions govern α-syn conformational changes and aggregation, which are thought to be involved in both α-syn biology and pathophysiology^5,6,10,11,56,57^.

We observe that α-syn-induced LD accumulation is consistent with the presence of higher levels of neutral lipid components. Interestingly, α-syn overexpression modulates both LD biosynthesis branches: TAG and CE synthesis. We reveal that α-syn causes a buildup in TAG due to DGAT2 upregulation and ACS activation, with no changes in TAG hydrolysis. Moreover, radiolabeling assays with [^3^H]-OA demonstrated not only increased acylation rates into TAG but also into DAG, accompanied by a rise in FA release. The rise in CEs was related to increased ACAT activation by α-syn overexpression. These results are complementary to a recent lipidomic analysis, which demonstrates that the expression of α-syn in different synucleinopathy models changes the cellular lipid profile, with a rise in DAG and TAG, dependent on the levels of α-syn. Moreover, in this same paper the lack of DGAT orthologous genes in yeast and the depletion of DGAT1 and DGAT2 in rat cortical neurons enhanced α-syn toxicity^31^, thus inferring that TAG accumulation protects against α-syn. This is in agreement with previous findings from our lab underpinning the protective role of TAG biosynthesis during overexpression of the mutant α-syn form A53T. In experiments in which TAG biosynthesis is pharmacologically inhibited and the consequent LD storage of FAs is prevented, neurons become more sensitive to A53T α-syn overexpression^30^. Moreover, genetic and chemical intervention of lipins modulates α-syn toxicity in yeast and mammalian cells^58^.

We detected higher levels of free chol in cells overexpressing α-syn. Our observations suggest that chol biosynthesis is not responsible for the chol increase despite the nuclear translocation of the transcription factor SREBP-2. While α-syn promotes the activation of SREBP-2, the classic effect on target genes involved in chol biosynthesis is not produced: α-syn overexpressing cells present lower HMGCR levels and no changes in DHCR24 levels. The same transcriptional response was found in a neurotoxic in vivo model of PD. The active form of SREBP-2 increases in the midbrain of mice treated with MPTP alongside downregulation of some canonical downstream genes, such as HMGCR^59^. Other lines of evidence suggest that α-syn aggregation is promoted under cell nutrient deprivation in response to ER stress, triggering SREBP-2 activation and subsequent cholesterolgenesis^60^. Moreover, α-syn aggregation is ameliorated when SREBP-2 activity is downregulated by means of chol synthesis reduction. Taking into account that SREBP-2 transcriptional activity has been associated with the expression of autophagy genes^51,52^, we demonstrate that α-syn overexpression causes ATG5 upregulation. Lipophagy is a known process implicated in LD breakdown^61^. In fact, when autophagy is blocked, cells overexpressing α-syn accumulate higher amounts of neutral lipids. Although lipophagy is active in our experimental model, our results suggest that the balance favors LD biogenesis over LD breakdown.

It has been proposed that chol dyshomeostasis in PD leads to increased levels of α-syn through alterations in lysosomal activity and other processes but that it bears no relation to direct α-syn-chol interactions^62^. Chol lysosomal accumulation was also observed playing a dual role in a cellular MPTP model of PD: protecting against lysosomal membrane permeabilization and cellular death and enhancing α-syn accumulation^63^. In this connection, our findings reveal that chol lysosomal accumulation is enhanced by WT α-syn. As we found that α-syn overexpression did not alter neither the lysosome distribution or the expression of lysosomal-specific markers, it could be hypothesized an impairment of chol trafficking. This latter could explain that chol avoids ER transport, thus altering lipid sensing and finally resulting in SREBP-2 nuclear translocation in cells overexpressing WT α-syn. However, we cannot discard that chol rise could be due to an increased chol uptake mediated by α-syn. There is a significant body of evidence, though mired in controversy, connecting changes in chol levels with PD^6,62,64^. Additionally, mutations in genes that encode lysosomal proteins are important genetic risk factors for developing PD. Among them, mutations in genes encoding the lysosomal proteins glucocerebrosidase (GBA1 gene) and sphingomyelinase (SMPD1 gene), faulty in Gaucher’s and Niemann-Pick diseases, respectively, have been connected to a higher predisposition to develop PD^65^. Further studies should be carried out to understand the role of chol lysosomal accumulation in synucleinopathies.

**Fig. 8:**
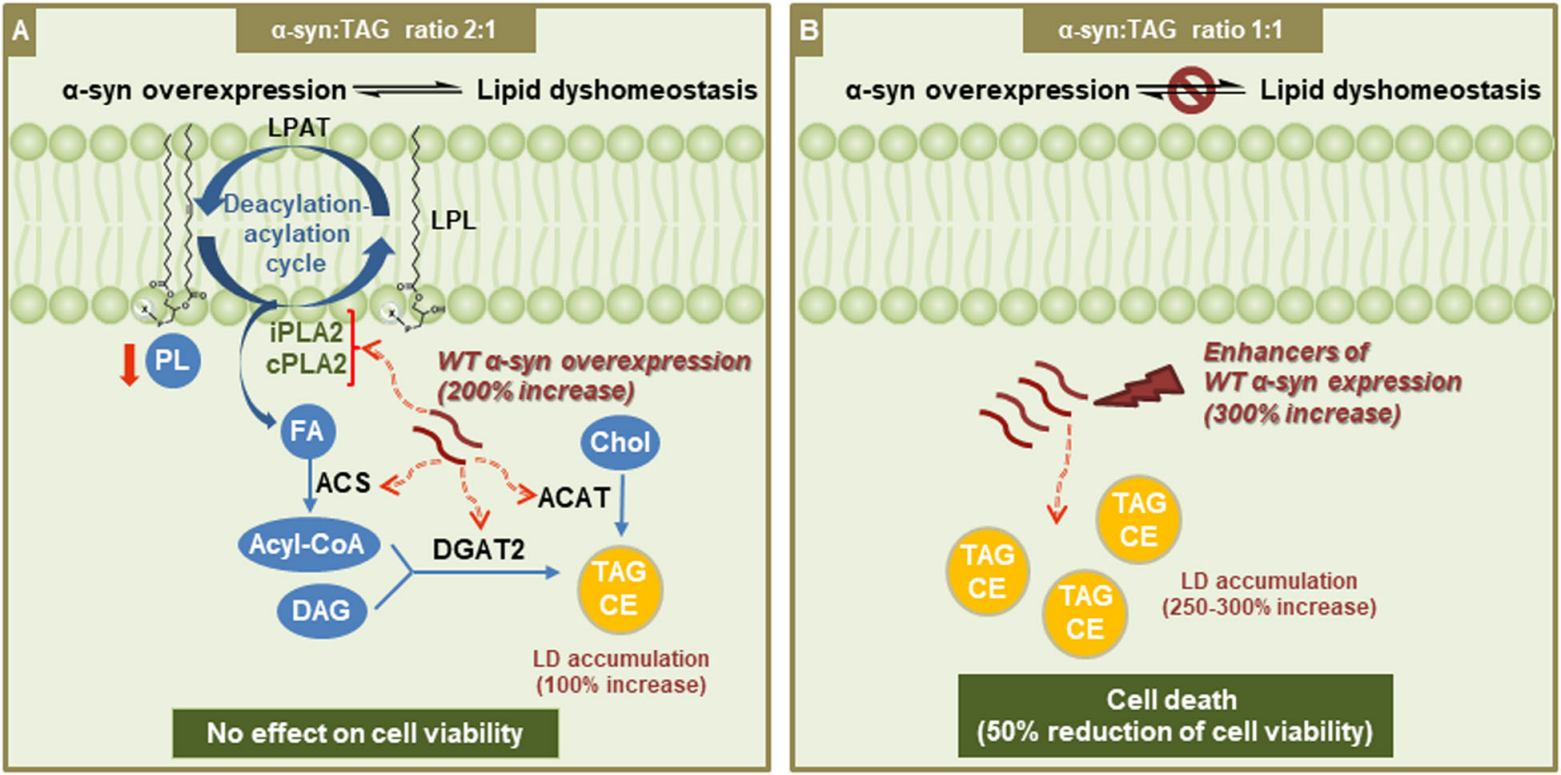
Schematic diagram showing impairment of lipid homeostasis triggered by α-syn and implications for cellular fate. **A** Imbalance of the PL deacylation-acylation cycle due to PLA2 activation mediated by α-syn overexpression is responsible for neutral lipid acylation and the consequent LD accumulation (α-syn:TAG ratio 2:1), with no changes in cell viability. **B** Under conditions of enhancement of α-syn expression, LD accumulation is exacerbated (α-syn:TAG ratio 1:1) and cell death is promoted.

Prevention of FA-mediated lipotoxicity is one of the mechanisms that activate LD formation under cellular stress^29^. A key role of OA in α-syn toxicity has been demonstrated using various models of α-syn dyshomeostasis^31^. Accordingly, the inhibition of OA production targeting stearoyl-CoA desaturase prevents α-syn toxicity and neurodegeneration. In contrast to our previous studies in which we demonstrated increased levels of FAS in neurons overexpressing A53T α-syn^30^, here we find FAS downregulation accompanied by lower activation of SREBP-1 in cells overexpressing WT α-syn. Therefore, our model shows that FA de novo synthesis does not contribute to higher FA bioavailability, thus indicating that lipid homeostasis disturbance occurs by different mechanisms depending on the α-syn variant. Our main contribution to the elucidation of the mechanisms involved in lipid homeostasis changes triggered by α-syn relates to the increase in TAG content as a consequence of the impairment of the PL deacylation-acylation cycle. WT α-syn overexpression induces the activation of iPLA2 and cPLA2 with an increase in FFA and a reduction in PL levels, and the consequent TAG buildup, thus preventing cell death. In line with our findings, it has been demonstrated that PLA2G6 knock-out mice exhibited an increase in PE and some species of PC levels in spinal cord samples^66^. In contrast, others found that the loss of iPLA2-VIa did not alter PL composition in fly mutants^67,68^. Additionally, our radiolabeling experiments showed an active turnover of FA in PA and PC. Hence, α-syn overexpression causes a dysregulation of the PL deacylation-acylation cycle providing FAs, whose fate is to be esterified into TAG (Fig. 8). During iPLA2 and cPLA2 inhibition, TAG accumulation is reduced and cells become more vulnerable to α-syn overexpression, thus revealing a novel protective strategy against α-syn toxicity. We propose that the concerted action of iPLA2 and cPLA2, and probably LPL hydrolases, could be a mechanism responsible for the decrease in PA and PC content in WT α-syn cells. iPLA2 deficiency has been implicated in the exacerbation of α-syn expression and aggregation^68,69^. iPLA2-VIA loss provokes the shortening of PL acyl chains in *Drosophila*. The consequent abnormalities in membrane composition not only promote ER stress and affect neurotransmission but also facilitate α-syn aggregation^68^. PLA2G6 knock-out mice exhibited increased expression of α-syn associated with mitochondrial damage^69^. The cellular response observed in our model by means of α-syn-induced PLA2 activation is LD accumulation, that might constitute a strategy to avoid α-syn/FA binding, a known mechanism of pathological oligomerization.

We report different experimental strategies using enhancers of α-syn expression showing potentiation of LD accumulation. In the scenarios of treatment with Btz, Mn and OA, the deleterious effects are more pronounced in cells overexpressing α-syn. Our work provides evidence that α-syn overexpression impairs lipid metabolism, leading to LD accumulation *via* PL deacylation (Fig. 8). The protective role of LDs is necessary for neuronal survival under moderate α-syn expression. However, under conditions that promote an increase in α-syn levels, LD biosynthesis is overwhelmed, rendering cells more vulnerable and diverting neuronal fate to death. In conclusion, our study indicates that lipid homeostasis impairment as evidenced in neutral lipid accumulation could be considered as an early marker of neurodegeneration that coexists in a steady-state with α-syn overexpression; however, when a threshold is crossed, cell viability is compromised (Fig. 8). These studies provide additional evidence for identifying novel therapeutic targets related to neutral lipid metabolism in neurodegenerative processes associated with α-syn overexpression.

## Supplementary information

Supplementary Figure Legend

Figure S1
