# Supplementary material for: Neutral lipids as early biomarkers of cellular fate: the case of α-synuclein overexpression
Source: Cell Death Dis. 2021 Jan 7;12(1):52. doi: 10.1038/s41419-020-03254-7 (PMC7791139; doi:10.1038/s41419-020-03254-7)
Supplement: Supplementary file 1 — Supplementary Figure Legend [file 41419_2020_3254_MOESM1_ESM.docx]

**Supplementary Figure Legends**

**Fig. S1. Lipophagy is involved in LD accumulation during α-syn overexpression. A.** Atg5 upregulation in cells overexpressing α-syn was determined by RT-qPCR. **B.** Alterations in LC3B in our model were visualized using immunocytochemistry. Hoechst was used as nuclear marker. **C.** The involvement of lipophagy in neutral lipid accumulation was analyzed by Oil Red O spectrophotometric measurement under autophagy inhibition using chloroquine (CQ) in WT α-syn cells. **D.** The effect of autophagy blockage by CQ on cell viability was determined by MTT reduction assay. **A-D.** *Scale bars* 20 µm. All experiments were repeated three times. Bars represent means ± standard deviation (SD, *n* = 3). **p* < 0.05, ***p* < 0.01, ****p* < 0.001 vs control.
