# Supplementary figures and images for: Neutral lipids as early biomarkers of cellular fate: the case of α-synuclein overexpression
Source: Cell Death Dis. 2021 Jan 7;12(1):52. doi: 10.1038/s41419-020-03254-7 (PMC7791139; doi:10.1038/s41419-020-03254-7)

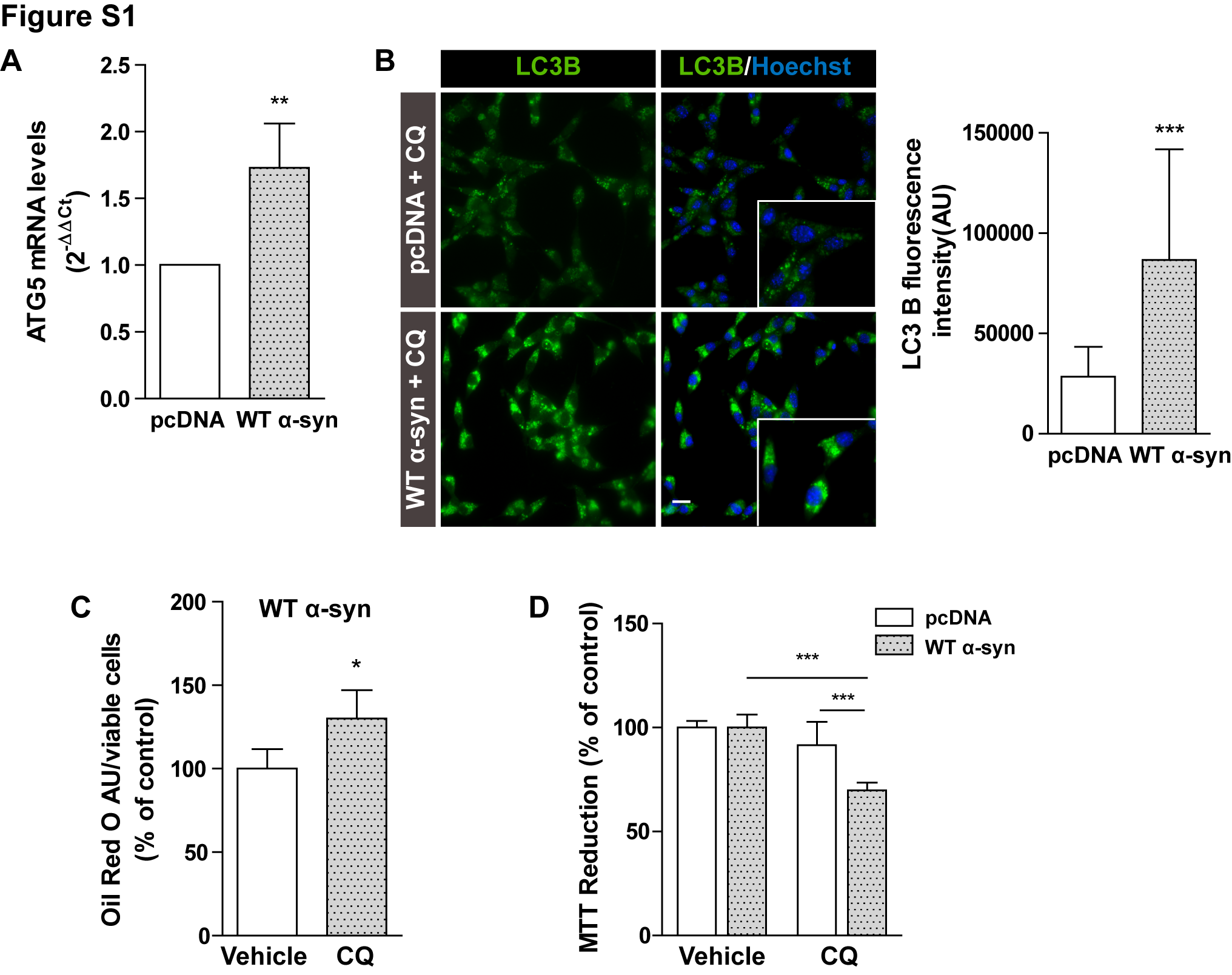

Supplement: Supplementary file 2 — Figure S1 [file 41419_2020_3254_MOESM2_ESM.tif]
